# Integrity of p53 Associated Pathways Determines Induction of Apoptosis of Tumor Cells Resistant to Aurora-A Kinase Inhibitors

**DOI:** 10.1371/journal.pone.0055457

**Published:** 2013-01-31

**Authors:** Yoshimi Shionome, Li Yan, Song Liu, Toshiaki Saeki, Toru Ouchi

**Affiliations:** 1 Department of Medicine, National University of Health Sciences, Pritzker School of Medicine, The University of Chicago, Evanston, Illinois, United States of America; 2 Department of Biostatistics and Bioinformatics, Roswell Park Cancer Institute, Buffalo, New York, United States of America; 3 Department of Breast Oncology, Saitama International Medical Center, Hidaka, Saitama, Japan; 4 Department of Cancer Genetics, Roswell Park Cancer Institute, Buffalo, New York, United States of America; Vanderbilt University Medical Center, United States of America

## Abstract

We have previously shown that mammary tumorigenesis in MMTV-Aurora-A mice is further enhanced when p53 is inactivated, demonstrating that integrity of p53 pathway determines phenotypes induced by this oncogenic kinase. In this study, we investigated the roles of genes involved in p53 pathway (p53, Puma, p21, Bax, and Chk2) in response to Aurora-A inhibitors, VX680 and MK-8745, and explored whether chemoresistant tumor cells would further undergo apoptosis with other therapeutic agents. Isogenic HCT116 cell lines were treated with VX680 or MK-8745. Cell cycle analysis, apoptosis, and tumorigenesity were studied. Chemoresistant cells were recovered from xenograft, and further induction of apoptosis was studied. Induction of apoptosis and aneuploidy with VX680 is much stronger than MK-8745. Xenograft assay indicates that tumor growth of HCT116 and HCT116 p53(-) cells are strongly inhibited by VX680, while that of other cell types are similarly inhibited by two compounds. Among the established cell lines recovered from xenografts, MK-8745-resistant clones contain elevated phosphorylation of mTOR and Akt. When further treated with inhibitors of both mTOR and Akt, those cells undergo apoptosis. These results indicate that p53-associated pathway plays a crucial role in regulating growth inhibition of tumor cells when treated with Aurora-A inhibitors. Combined treatment with Akt/mTOR inhibitors can further induce apoptosis of Aurora-A tumors.

## Introduction

Aurora-A kinase is frequently overexpressed in varieties of human cancers and cancer cell lines, and can transform fibroblasts when transfected [1]–[7]. We have recently generated transgenic mice model expressing MMTV-Aurora-A, in which mammary tumors are induced after relatively long latency (∼2 years) [8]. In this mice model, tumor incidence is enhanced when one allele of p53 is deleted, suggesting that integrity of p53 pathway determines tumor progression of mammary tumors in these mice, although functional interaction between p53 pathway and Aurora-A tumorigenesis remains to be detailed. These results clearly indicate strong evidence that Aurora-A functions as an onco-protein.

In our MMTV-Aurora-A model, immunohistochemical analysis of tumors developed in these mice show that Akt and mTOR are activated [8]. Given the accumulating evidence that Akt and mTOR pathway is closely associated with cell proliferating and transformation, it is suggested that Aurora-A and Akt/mTOR cooperate in mammary carcinogenesis. On the basis of these observations, we generated evidence that these two pathways can collaborate for cell transformation in vitro. In those experiments, although transient overexpression of Aurora-A does not induce phosphorylation of Akt/mTOR immediately, phosphorylation of these proteins appears after prolonged culture of Aurora-A overexpressing cells [9]. Significantly, only Akt/mTOR-activated cells, but not immediate Aurora-A transfectants, show accelerated colony forming abilities, supporting a model that co-activation of Akt/mTOR is necessary for malignant phenotypes of Aurora-A positive tumors, supporting the previous studies of Akt regulation by Aurora-A [10], [11].

It has been well illustrated that treatment of cancer transformed by oncogenic kinases with small kinase inhibitors results in successful outcome [12]–[14], although more detailed analyses of biochemical and biological properties of each of the inhibitors need to be studied. VX680 was synthesized as a prototype of an Aurora-A inhibitor and strongly inhibits tumor growth in vitro as well as in vivo [15]. MK-8745 is a novel Aurora-A inhibitor which has more recently been developed, and induces significant growth arrest of natural killer (NK) cell lymphoma [16]. In the current studies, we used human colon cancer cell line, HCT116, in which Aurora-A is amplified, and its isogenic derivatives in which p53, p21, Puma, Bax and Chk2 are stably knocked out [17]–[20]. Since our previous data indicates that p53 pathway is involved in determination of malignant phenotypes induced by Aurora-A, we investigated the roles of p53-associated proteins by taking advantage of these isogenic cell lines. Series of xenograft assay using these cells with chemical inhibitors would demonstrate how p53 pathway determines tumor cells' sensitivities when treated with VX680 andMK-8745. In the current studies, we also explored tumor growth and biochemical analysis of chemoresistant clones recovered from xenograft and examined whether combinational treatment of these cells with inhibitors of Aurora-A, mTOR and Akt could cooperate in tumor suppression. Pre-clinical research shown here will provide us with better and potential strategies targeting Aurora-A tumors.

## Materials and Methods

### Ethics statement

We certify that mice were treated in accordance with the guidelines of University of Chicago (Evanston, USA). A protocol of mice studies was approved by Northshore University Health System IACUC. When tumor size reaches 1.5 cm, tumors were be removed and mice were euthanized by CO_2_ asphyxiation followed by cervical dislocation.

### Cell culture

HCT116 was purchased from ATCC and isogenic HCT116 variants deficient for p53, Puma, Bax, Chk2 or p21 were kindly obtained from Dr. Bert Vogelstein (Johns Hopkins University, Ref. 17–20). They were grown in McCoy's 5A medium supplemented with 10% fetal bovine serum and 100 U of penicillin-streptomycin/ml (Invitrogen). HCT116 variants recovered from xenograft were also maintained in the same condition.

### Cell cycle analysis of isogenic HCT116 variants when treated with kinase inhibitors

VX680 and MK-8745 were obtained from Merck Inc. on the basis of material transfer agreement (both stock solution is 1 mM, respectively). mTOR inhibitor Pp242 and Akt inhibitor VIII were purchased from Chemdea. Neocarzinostatin was purchased from KAYAKU (Japan). Cells were treated with VX680 (800 nM) or MK-8745 (800 nM) for 48 h, or Pp242 (8 nM) and VIII (210 nM) for 24 h, or neocarzinostatin (0.5 µml) for 12 h. After treatment, cells were harvested, trypsinized and fixed in 70% ethanol overnight at −20°C. Cells were washed with PBS, treated with RNase for 30 min, followed by staining with propidium iodide. The DNA content was analyzed with FACSCalibur and CellQuest Pro software. Results were obtained from at least five independent experiments.

### Xenograft assay

All animal studies were performed by following protocol approved by Institutional Animal Care and Use Committee (IACUC). Female athymic mice at the age of 4 to 5 weeks were purchased from Jackson Laboratory. For xenograft assay, HCT116 isogenic cells (5×10^6^ cells/100 µl of PBS) were transplanted into both flanks of mice. When tumor size reached at 200∼400 mm^3^, one side of tumor was s.c. injected with VX680 (800 nM) or MK-8745 (800 nM). Size of tumors was measured every other day after injection. Tumor volume (v) was determined using the following equation: v = (width)^2^×length/2. Relative tumor volume was calculated as follows: (Vi-V1)/V1×100, where V1 and Vi indicate tumor volume at day1 and day i after drug treatment, respectively. When tumor size reached to 2,000 mm^3^, mice were euthanized. Five mice were used for one isogenic HCT116 variants, and experiments were repeated at least three times.

### Removal and re-xenograft of drug resistant tumors

After administration of VX680 or MK-8745 for tumors developed in nude mice for 7 to 12 days, chemoresistant tumors were excised and placed on cell culture plate in McCoy's 5A medium supplemented with 10% fetal bovine serum and 100 U of penicillin-streptomycin/ml, as described above. On the next day, VX680 was added to cell culture media (400 nM or 800 nM) of VX680 resistant cells, and similarly MK-8745 was added to MK-8745 resistant cell culture (400 nM or 800 nM), until cell colonies were isolated. Of note, VX680 resistant clone was obtained only from HCT116 p21(-) tumor, although MK-8745 resistant clones were obtained from HCT116 Puma(-), p53(-), Chk2(-), Bax(-) and p21(-) cells. Isolated cells were maintained in the presence of 100 nM of either VX680 or MK-8745, and 5×10^6^ cells were transplanted again into nude mice as described above.

### Western blot analysis

Cells were lysed with EBC buffer (50 mM Tris-HCl, pH 8.0, 120 mM NaCl, 0.5% NP-40, 100 mM NaF, 200 nM sodium orthovanadate, 100 µg/ml phenylmethysulfonyl fluoride, 2 µg/ml leupeptin, 2 µg/ml aprotinin). Protein concentration was determined by Bradford method, followed by separation in 5% or 7.5% sodium dodecyl sulfate-polyacrylamide gel electrophoresis (SDS-PAGE). Proteins were transferred to Immobilon-P membrane (Milipore) using a semidry transfer method (Trans-Blot, Bio-Rad). Membranes were blocked in 1% nonfat dried milk and 0.05% Tween 20 in PBS. Primary antibodies are: anti p53, Akt1/2, anti β-actin (Santa Cruz), anti anti-Aurora-A, phospho-Akt Ser473, mTOR, phospho-mTOR Ser2448, phosphor-mTOR Ser2481 (Cell Signaling). Secondary antibodies are horseradish peroxidase-conjugated anti mouse IgG or rabbit IgG (Jackson Laboratory).

## Results

### Differential regulation of the cell cycle by VX680 and MK-8745 Aurora-A inhibitors

**Figure 1:**
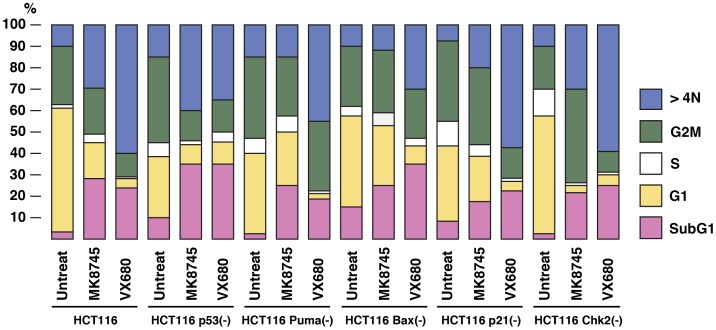
The effects of VX680 or MK-8745 on cell cycle. HCT116 and their isogenic variants, HCT116 p53(-), HCT116 Puma(-), HCT116 Bax(-) HCT116 p21(-) and HCT116 Chk2(-), were treated with VX680 (800 nM) or MK8734 (800 nM) for 48 h. Experiments were repeated in at least five independent experiments. Statistical analysis was determined by the Student's *t*-test (p<0.05).

To study the roles of p53 pathway in Aurora-A tumorigenesis, we used HCT116 human colorectal cancer cell line overexpressing Aurora-A [21], and its isogenic derivatives, in which p53-associated genes (p53, p21, Puma, Bax and Chk2) are stably knocked out in vitro (17–20). Logarithmically growing cell culture of these variants was treated with VX680 (800 nM) or MK-8745 (800 nM) for 48 h, and cell cycle profiles were determined by FACS analysis (Figure 1). VX680 induced apoptosis of HCT116 (23%), HCT116 p53(-) (35%), HCT116 Bax(-) (32%), HCT116 p21(-) (21%) and HCT116 Chk2(-) (25%) cells, but less in HCT116 Puma(-) cells (17%). VX680 also induced aneuploidy significantly in HCT116, HCT116 Puma(-), HCT116 p21(-) and HCT116 Chk2(-) cells, but it was much less in HCT116 Bax(-) cells. MK-8745 induced apoptosis of HCT116 (27%), HCT116 p53(-) (35%), HCT116 Puma(-) (25%), HCT116 Bax(-) (25%), and HCT116 Chk2(-) (22%) cells, but less in HCT116 p21(-) cells (15%). Induction of aneuploidy by MK-8745 was high in HCT116 p53(-) cells (40%), or intermediate in HCT116 and HCT116 Chk2(-) cells (30% and 33%, respectively), but much less in HCT116 Puma(-), HCT116 Bax(-) and HCT116 p21(-) cells. These results indicate that both VX680 and MK-8745 can potentially induce apoptosis, but aneuploidy is well induced by VX680, compared to that of MK-8745.

### Differential tumorigenesis of HCT116 variants

**Figure 2:**
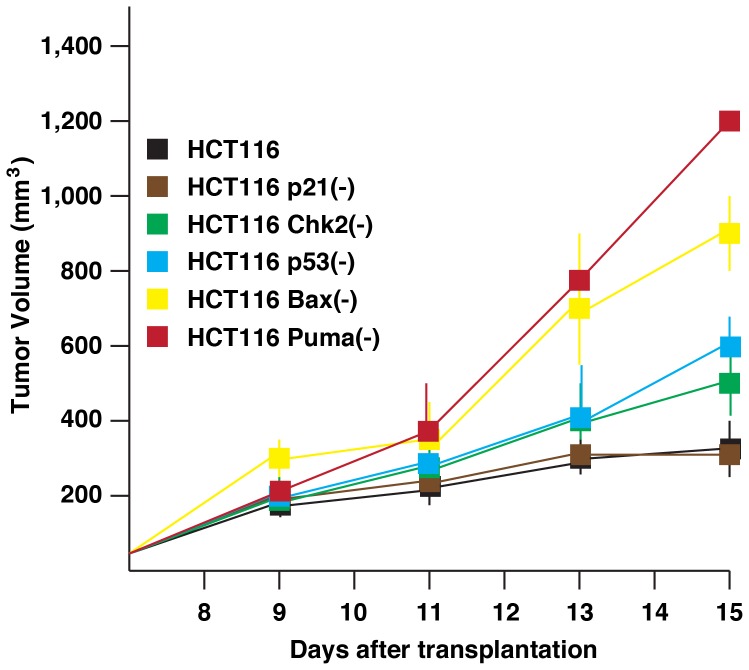
Tumorigenesis of HCT116 isogenic variants in nude mice. Indicated HCT116 cells (5×10^6^ cells) were transplanted into nude mice. Size of tumors was measured every other day. Five nude mice per cell type were used, and experiments were repeated at least three times. Statistical analysis was determined by the ANOVA analysis.

We next detailed anti-tumor activity of VX680 and MK-8745 by xenograft assay of HCT116 cells using nude mice. First, tumorigenesis of these HCT116 isogenic cells was determined without providing VX680 and MK-8745. Cells (5×10^6^ cells/injection) were transplanted to female athymic mice (4 to 5 weeks age). Tumors became palpable on day 7 in all of these cell lines studied, and a size of these tumors was measured every other day for the next 8 days (Figure 2). Parental HCT116 and HCT116 p21(-) showed similar tumor growth in these 15 days after transplantation. We performed the ANOVA analysis and found significant differences in all comparisons below; compared to these two cell lines, HCT116 p53(-) and HCT116 Chk2(-) cells showed faster tumor growth, and HCT116 Puma(-) and HCT116 Bax(-) cells showed much aggressive tumor growth. These results clearly indicate that inactivation of Puma or Bax accelerates tumorigenesis.

**Figure 3:**
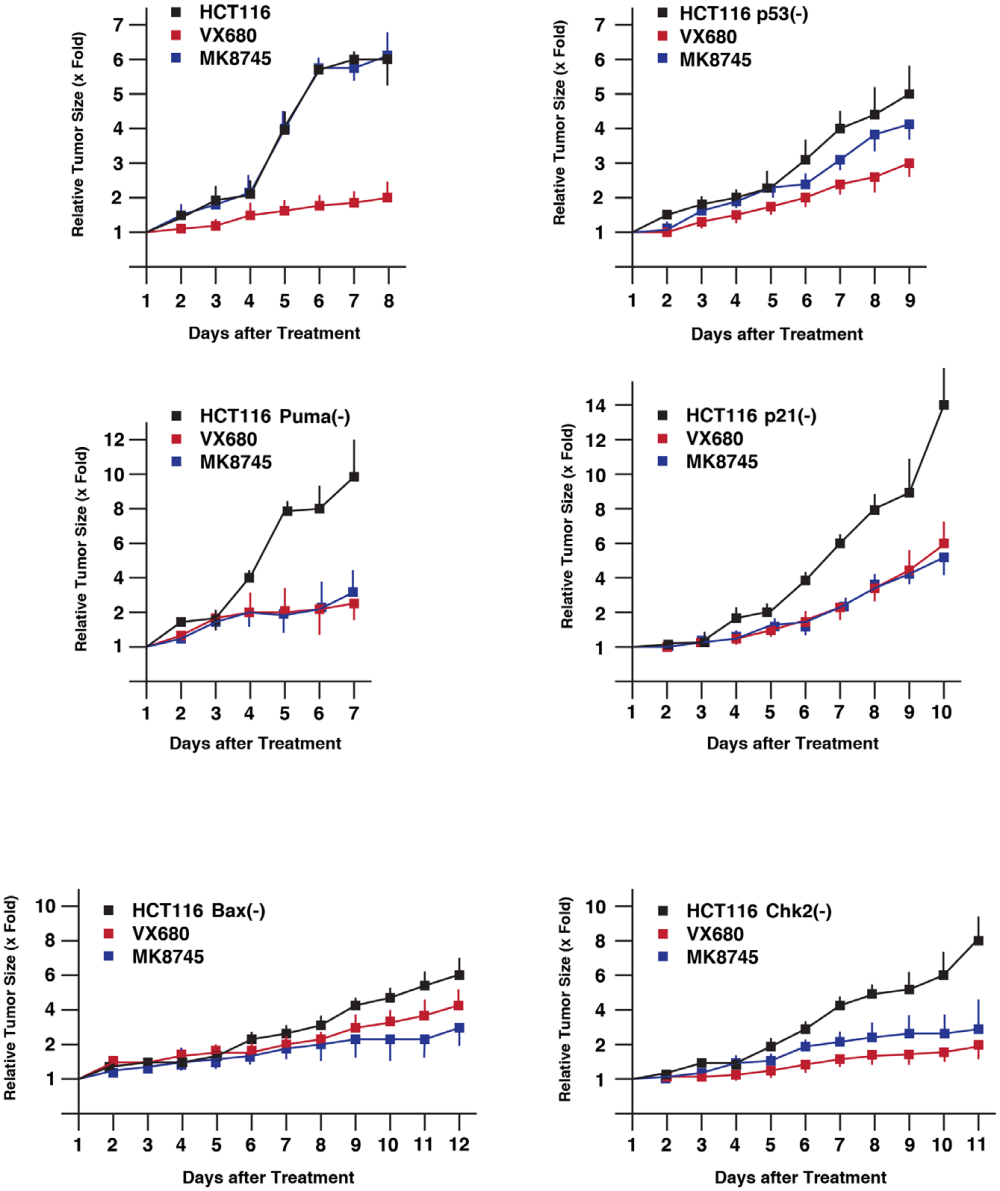
Anti-proliferative effects of VX680 or MK-8745 in vivo. Each of the indicated HCT116 variants was transplanted into both flanks of a mouse. When tumor size reached at 200–400 mm3, VX680 or MK-8745 (800 nM) was injected into one of the developed tumors per mouse. Tumor size was measured every day. Total five mice were used for these studies in two independent experiments. Statistical analysis was determined by the 2 way ANOVA analysis.

We then explored anti-tumor activity of VX680 and MK-8745 by xenograft model (Figure 3). HCT116 isogenic cell lines were transplanted onto athymic mice, and a size of tumor was measured every other day as described above. When the size became ∼200 to 400 mm^3^, VX680 (800 nM) or MK-8745 (800 nM) was directly s.c. injected daily. Tumor growth of HCT116, HCT116 p53(-), HCT116 Puma(-), HCT116 p21(-) and HCT116 Chk2(-) cells was significantly reduced by VX680, but inhibition of HCT116 Bax(-) cells was much less than the other cell types. Inhibition of tumor growth of HCT116 cells with MK-8745 was not significant, and HCT116 p53(-) tumorigenesis was weakly inhibited with this compound. Compared to these two cell types, tumor growth of HCT116 Puma(-), HCT116 p21(-), HCT116 Bax(-) and HCT116 Chk2(-) cells was significantly inhibited with MK-8745. By using SAS 9.3, 2 way ANOVA analysis was conducted for the post hoc analysis on the tumor size fold changes using Tukey's studentized range test (HSD) on all main effect mean. The time points when the 3 groups (untreated, treated with VX680, treated with MK8745) show significant differences is: HCT116 cells; at day 4, p53(-) cells; at day 3, Puma(-) cells; at day 4, p21(-) cells; at day 6, Bax(-) cells; at day 12, Chk2(-) cells; at day 7.

These results demonstrate that anti-tumor activity of VX680 is p53-independent, and it is less effective when Bax is inactivated. MK-8745 did not show significant tumor inhibition of HCT116 and HCT116 p53 cells, however, inactivation of Puma, p21, Bax and Chk2 could enhance its anti tumor activity.

### VX680- or MK-8745-resistant tumor cells do not show enhanced tumorigenecity

Xenograft experiments indicated that VX680 and MK-8745 show anti-tumor activity, however, they did not regress tumors completely, and drug-resistant tumors still remained in mice. We characterized whether these drug-resistant cells show more malignant phenotypes than those of the individual original HCT116 variants. After treatment of VX680 or MK-8745, tumors were removed from xenograft of HCT116, HCT116 p53(-/-), HCT116 p21(-/-), HCT116 Puma(-/-), HCT116 Bax(-/-), and HCT116 Chk2(-/-), excised and seeded on cell culture plates. Tumor cells resistant to VX680 were maintained in the presence of VX680 (400 nM), and those resistant to MK-8745 were maintained in the presence of MK-8745 (800 nM) to establish drug-resistant subclones. Among the removed tumors, VX680-resistant cell lines of HCT116 Puma(-/-), and MK-8745-resistant cell lines of HCT116 Puma(-/-), HCT116 Bax(-/-), HCT116 p53(-/-), HCT116 p21(-/-) and HCT116 Chk2(-/-) were successfully established.

**Figure 4:**
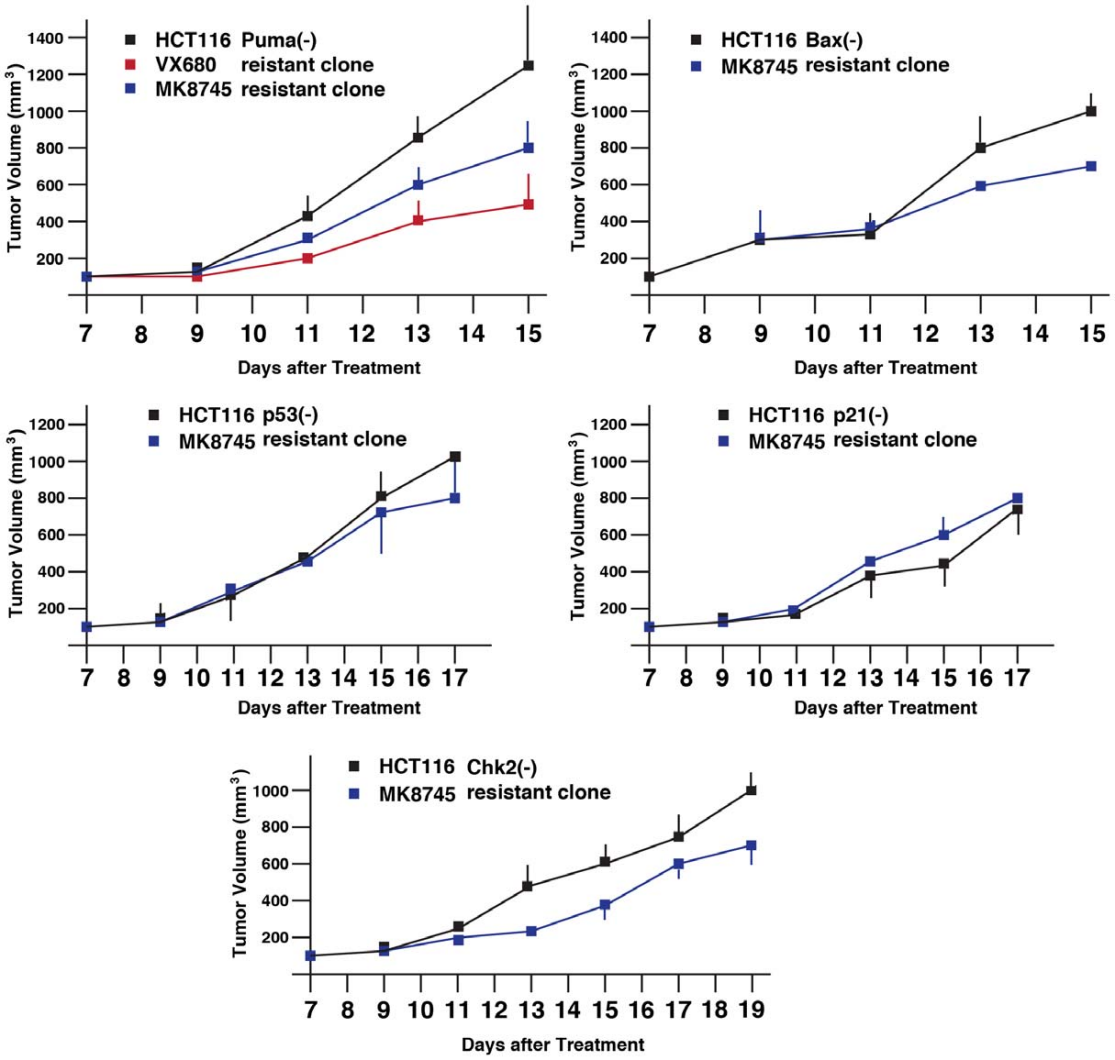
Tumor growth of HCT116 variants resistant to either VX680 or MK-8745. Parental and VX680/MK-8745-resistant HCT116 Puma(-) cells, parental and MK-8745-resistant HCT116 Bax(-) cells, parental and MK-8745-resistant HCT116 p53(-) cells, parental and MK-8745-resistant HCT116 p21(-) cells, parental and MK-8745-resistant HCT116 Chk2(-) cells were transplanted into nude mice. Size of developed tumors was monitored every other day. Five mice per cell type were used, and experiments were repeated at least two times. Statistical analysis was determined by the 2 way ANOVA analysis.

Next, tumor xenograft assay was performed to study whether these recovered cell lines show more malignant phenotypes than each of the original cell lines (Figure 4). As described above, 5×10^6^ cells were transplanted onto nude mice, and a size of the developed tumors was daily measured from day 7.

MK-8745-resistant clones of HCT116 Puma(-/-), HCT116 Bax(-/-), HCT116 p53(-/-), and HCT116 Chk2(-/-) cells showed slightly slow tumor development compared to each of the original control cells. In contrast, MK-8745-resistant HCT116 p21(-/-) cells showed slightly faster tumorigenesis than their control cells.

Interestingly, tumor growth of VX680-resistant HCT116 Puma(-/-) cells were much slower than their parental HCT116 Puma(-/-) cells.

Using SAS 9.3, two way ANOVA analysis has been conducted for the post hoc analysis on the tumor size changes, using Tukey's studentized range test (HSD) on all main effect mean. The results indicate the time points when differences between parental and resistant cells are observed are: HCT116 Puma(-) and VX680/MK8745 cells; at day 15, HCT116 Bax(-) and MK8745 cells; at day 13, HCT116 p53(-) and MK8745 cells; at day 15, HCT116 p21(-) and MK8745 cells; at day 13, HCT116 Chk2(-) and MK8745 cell; at day 15.

These results indicate that drug-resistant tumors did not acquire accelerated tumorigenesis. Rather, these cells demonstrated lowered tumor growth in vivo, when re-transplanted into mice. It remains to be elucidated how these cells escaped from apoptosis and aneuploidy after treatment with VX680 or MK-8745, as indicated in Figure 1B.

### Akt and mTOR are activated in VX680- and MK-8745-resistant tumors

**Figure 5:**
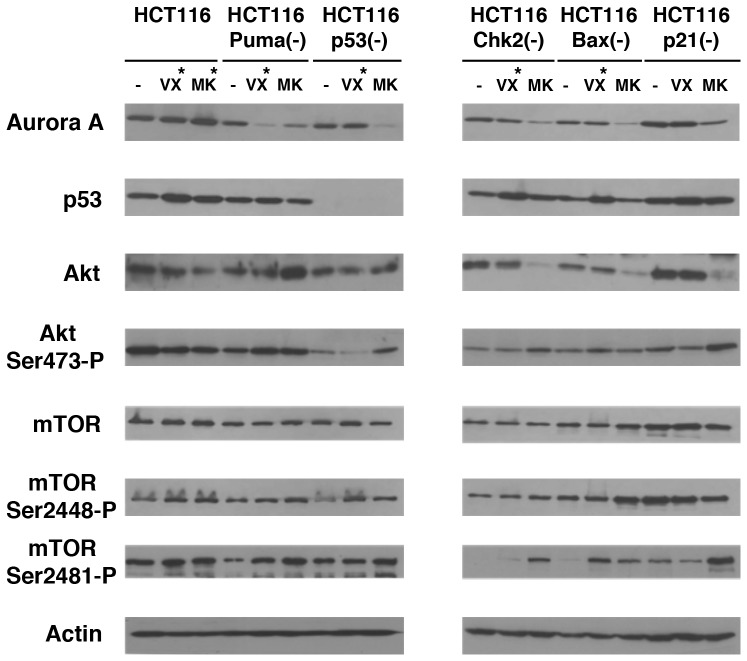
Phosphorylation of mTOR and Akt is elevated in chemoresistant tumors. Levels of indicated proteins and their phosphorylation were studied in MK-8745-resistant HCT116 Puma(-), p53(-), Chk2(-), Bax(-), p21(-) cells, and VX680-resistant HCT116 p21(-) cells. As references (indicated with *), HCT116 cells were transiently treated for 24 h with VX680 or MK8734, and HCT116 Puma(-), p53(-), Chk2(-), Bax(-) and p21(-) cells were transiently treated with VX680 for 24 h. Those cells were also immunoblotted.

Our previous studies have demonstrated that Akt and mTOR are constitutively phosphorylated in mammary tumors developed in MMTV-Aurora-A mice, suggesting that activation of Akt/mTOR is involved in Aurora-A's cell transformation [8], [9]. We studied phosphorylation of Akt at Ser473, mTOR at Ser2448 and Ser2481 in drug-resistant HCT116 variants established from tumors (Figure 5). Since VX680-resistant clones of HCT116, HCT116 Puma(-), HCT116 p53(-), HCT116 Chk2(-) and HCT116 Bax(-) cell lines could not be established from xenograft, original isogenic cell lines were treated with VX680 for 24 h and used for biochemical analyses. Similarly, MK-8745-resistant clones of HCT116 cell line could bot be established, cells were treated with MK-8745 for 24 h and used for biochemical analyses.

In all of the stable MK-8745-resistant clones, levels of endogenous Aurora-A were significantly decreased. Reduced levels of Akt were observed in MK-8745-resistant clones of HCT116 Chk2(-), HCT116 Bax(-) and HCT116 p21(-) clones, and increase in Akt Ser473 phosphorylation was observed in HCT116 p53(-), HCT116 Chk2(-), HCT116 Bax(-) and HCT116 p21(-) cells.

Levels of mTOR were not significantly changed in MK-8745-resistant clones, compared to those of each parental clone. Phosphorylation of mTOR at Ser2448 was increased in MK-8745-resistant HCT116 Puma(-), HCT116 p53(-), HCT116 Bax(-) cells, and phosphorylation of mTOR at Ser1481 was increased in MK-8745-resistant HCT116 Puma(-), HCT116 p53(-), HCT116 Chk2(-), HCT116 Bax(-) and HCT116 p21(-) cell.

In the stable VX680-resistant HCT116 p21(-) cells, levels of Aurora-A, Akt and mTOR were comparable with its parental HCT116 p21(-) cells. Phosphorylation of Akt at Ser473, mTOR at Ser2448 and Ser2481 was also similar to those of the parental HCT116 p21(-) cells.

When HCT116 isogenic variants were treated with MK-8745 for 24 h, increased phosphorylation of mTOR at Ser2448 was observed in HCT116 cells. When treated with VX680 for 24 h, phosphorylation of mTOR at Ser2448 was increased in HCT116 and HCT116 p53(-) cells, and phosphorylation of mTOR ar Ser2481 was increased in HCT116 Bax(-) cells.

These results indicate that phosphorylation of either Akt and/or mTOR is closely associated with VX680- or MK-8745-resistant phenotypes. However, these drug-resistant clones did not show enhanced tumorigenesis in xenograft (Figure 4), suggesting that activation of Akt/mTOR is involved in anti-apoptotic phenotype of these cells rather than aggressive tumorigenicity.

### Inhibition of Akt and mTOR induces apoptosis of MK-8745-resistant cells

**Figure 6:**
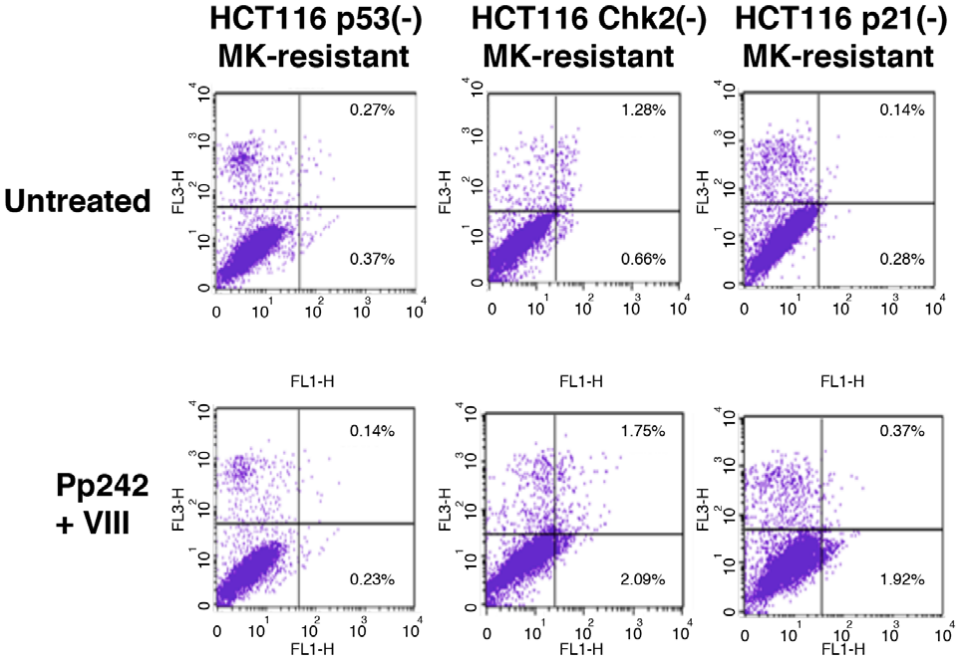
MK-8745-resistant HCT116 variants undergo apoptosis when treated with inhibitors of mTOR and Akt. MK-8745-resistant HCT116 p53(-), Chk2(-) and p21(-) cells were treated with Akt inhibitor VIII (210 nM) and mTOR inhibitor Pp242 (8 nM) for 24 h, followed by Annexin V staining. Statistical analysis was determined by the Student's *t*-test (p<0.05).

We have previously shown that mammary tumors developed in MMTV-Aurora-A transgenic contain phosphorylated U. Since our results indicate that Akt/mTOR is phosphorylated in drug-resistant clones, we hypothesized that activation of Akt/mTOR is involved in anti-apoptotic phenotypes of these cells. We used three stable MK-8745-resistant clones, HCT116 p53(-), HCT116 Chk2(-) and HCT116 p21(-) cell lines, since both mTOR and Akt are simultaneously phosphorylated in these cells. These cells were treated with Pp242 (mTOR inhibitor) and VIII (Akt inhibitor) for 24 h, and apoptosis was measured by Annexin V staining (Figure 6). Apoptosis of MK-8745-resistant-HCT116 Chk2(-) cells was induced from 1.94% to 3.84%, and that of MK-8745-resistant-HCT116 p21(-) cells was induced from 0.42% to 2.29%, respectively. Apoptosis of MK-8745-resistant-HCT116 p53(-) cells was not significantly induced under conditions of these treatment. These results support a notion that activation of Akt/mTOR is involved in anti-apoptosis of cells resistant to Aurora-A inhibitors, and that those cells can undergo apoptosis when Akt/mTOR activities are inhibited. We also studied how HCT116 isogenic cells resistant to Aurora-A inhibitors respond to ionizing mimetic anti-tumor agent, neocarzinostatin. Among the cell lines described in Fig. 4, HCT116 Chk2(-) cell underwent apoptosis (10.6%), and MK-8745-resistant HCT116 Chk2(-) cells further underwent apoptosis (20.3%) (Table S1). These results suggest that apoptotic sensitivities of Aurora-A inhibitor-resistant tumor cells to ionizing radiation could be determined by Chk2-associated pathway.

## Discussion

Aurora-A kinase regulates cell division by phosphorylating multiple downstream targets in mitotic machinery [22]–[24]. Of clinically importance is that this kinase is frequently overexpressed in varieties of human cancers [1]–[7]. Several in vitro experiments support that elevated expression of Aurora-A is oncogenic since transfection of fibroblasts with Aurora-A results in cell transformation [1]–[7]. These observations provide foundation that inhibition of this kinase could contribute to tumor suppression.

We have previously generated transgenic mice, in which Aurora-A is highly expressed in mammary gland. Although Aurora-A causes cell transformation rapidly in vitro, tumorigenesis in these mice is not frequent and its latency is quite long (∼2 years) [8]. These results strongly suggest that increased levels of Aurora-A are not an immediate driving force, but additional oncogenic pathway(s) needs to be activated for tumor development. In fact, immunohistochemistry analysis of mammary tumors developed in our mice indicated that phosphorylation of Akt and mTOR is increased, suggesting that activation of Akt/mTOR pathway collaborates with Aurora-A, leading to cell transformation [8], [9].

Roles of Akt/mTOR pathway in Aurora-A transformation have also been implicated from our and other's previous studies [9]–[14]. Intrinsic roles of Akt/mTOR pathway in cell proliferation have also been well illustrated previously [25], [26]. We discovered that Aurora-A cells contain phosphorylated Akt/mTOR demonstrated after long-term cell culture, compared to those in short term cell culture and that those Akt/mTOR active cells show much aggressive colony forming abilities than those without Akt/mTOR phosphorylation. Roles of p53 pathway in Akt/mTOR activation in Aurora-A's transformation are further demonstrated in the current studies. Thus, Akt and mTOR are phosphorylated in variants of HCT116 cell lines that are resistant to VX680 or MK-8745 in xenograft assay. Importantly, these cells undergo apoptosis when treated with Akt/mTOR inhibitors. These results suggest that combination therapy with inhibitors of Aurora-A, mTOR and Akt could inhibit Aurora-A tumor malignancy, although integrity of p53 pathway is crucial for induction of apoptosis.

Although our data indicates functional collaboration between Aurora-A and Akt/mTOR, the mechanism of how phosphorylation of Akt and mTOR is induced in tumors remains to be elucidated. It is possible that Akt/mTOR pathway become activated in the course of tumorigenesis and provides growth advantage to Aurora-A cells in vivo. However, it is also possible that original pool of Aurora-A cells are heterogeneous and that Akt and mTOR are already activated in a small fraction of cells in this pool. In that sense, these small numbers of Aurora-A positive cells containing activated Akt/mTOR could survive when VX680 or MK-8745 is provided into mice. This hypothesis that cells' sensitivities to Aurora-A inhibitors is determined by activities of Akt/mTOR is supported by our previous results indicating that Aurora-A inhibitors cause apoptosis only when Akt/mTOR pathway is not activated [9]. Nevertheless, most interestingly, these Akt/mTOR-activated cells that are resistant to VX680 or MK-8745 do not show aggressive tumorigenesis compared to each of their parental cells, when re-transplanted into mice. These results suggest that Akt/mTOR-activated cells could indicate a cancer stem cells phenotypes in Aurora-A tumors.

In this study, we used isogenic HCT116 cell lines in which p53-associated genes are deleted. These variants would help identify the pathway(s) that could determine cells sensitivity to Aurora-A inhibitors. Among the genes studied include p53, Puma, Bax, Chk2 and p21, which are involved in apoptosis, cell cycle and checkpoint. In cell culture experiments shown in Figure 1, both VX680 and MK-8745 induced significant apoptosis in all of the HCT116 variants examined, suggesting that these inhibitors cause apoptosis in p53-independent manner. Recent studies have shown that VX-680 suppresses Aurora-A-induced tumorigenesis at 100 nM in 96 h [15], however we could not see these effects with same concentration of this agent. This might be due to different cell culture conditions, therefore we used higher dose. We recognize that unknown kinases could also be inhibited in our experimental settings. Although recent studies by Nair et al. indicated that induction of polyploidy and apoptosis by MK-8745 is p53-depdendent [27], our data indicate that MK-8745 induces apoptosis in p53-independent manner. This difference might be due to our prolonged incubation time (40 h versus 48 h). Nevertheless, of much pre-clinical importance is that these cells demonstrated different sensitivities to Aurora-A inhibitors in xenograft in this study. These results illustrate that tumor microenvironment determine cell's sensitivities to chemotherapy. One of the unexpected results is increased sensitivities of HCT116 Bax(-) cells to VX680 and MK-8745 in xenograft. Given the pro-apoptotic activities of Bax [28], [29], it is possible that VX680 and MK-8745 use alternative apoptosis pathway in xenograft, although detailed mechanism is under investigation. Finally, we determined the genomic sequence of Aurora-A coding exons of HCT116 cells before and after treatment of VX680 or MK-87845. No mutations were identified after these treatments (data not shown), suggesting that acquisition of drug-resistance of HCT116 variants is not due to alteration of the Aurora-A protein structure.

In summary, our studies here demonstrate evidence that VX680 and MK-8745 could induce tumor suppression in vivo. When VX680/MK-8745-resistant tumors are further treated with Akt/mTOR inhibitors, those cells underwent apoptosis. These results would indicate a potentially new strategy to treat tumors overexpressing Aurora-A kinase.

## Supporting Information

Table S1 **Ionizing radiation mimetic neocarzinostatin induces apoptosis of HCT116 Chk2(-) and MK-8745-resistant HCT116 Chk2(-) cells.** HCT116 isogenic variants (Puma(-), Bax(-), p53(-), p21(-) and Chk2(-) cells) and their Aurora-A inhibitor-resistant variants recovered from xenograft (Figure 3) were further treated with neocarzinostatin (0.5 µg/ml, 12 h), and cell cycle profile was determined by at least two independent FACS analysis.
(DOCX)
