# Supplementary material for: Integrity of p53 Associated Pathways Determines Induction of Apoptosis of Tumor Cells Resistant to Aurora-A Kinase Inhibitors
Source: PLoS One. 2013 Jan 31;8(1):e55457. doi: 10.1371/journal.pone.0055457 (PMC3561291; doi:10.1371/journal.pone.0055457)
Supplement: Table S1 — Ionizing radiation mimetic neocarzinostatin induces apoptosis of HCT116 Chk2(-) and MK-8745-resistant HCT116 Chk2(-) cells. HCT116 isogenic variants (Puma(-), Bax(-), p53(-), p21(-) and Chk2(-) cells) and their Aurora-A inhibitor-resistant variants recovered from xenograft (Figure 3) were further treated with neocarzinostatin (0.5 µg/ml, 12 h), and cell cycle profile was determined by at least two independent FACS analysis. (DOCX) [file pone.0055457.s001.docx]

|  | SubG1 | G1 | S | G2M |
| --- | --- | --- | --- | --- |
| Puma(-) | 1.7 | 49.9 | 8.5 | 39.9 |
| Puma(-) + NCS | 2.3 | 24.9 | 3.3 | 69.5 |
| Puma(-) MK-R | 2.3 | 56.8 | 3.0 | 37.9 |
| Puma(-) MK-R + NCS | 2.7 | 37.2 | 12.7 | 47.4 |
| Puma(-) VX-R | 2.6 | 58.6 | 4.5 | 34.3 |
| Puma(-) VX-R + NCS | 4.3 | 36.5 | 1.9 | 57.3 |
| Bax(-) | 2.8 | 47.3 | 6.6 | 43.3 |
| Bax(-) + NCS | 3.1 | 17.8 | 1.8 | 77.3 |
| Bax(-) MK-R | 3.1 | 51.6 | 6.5 | 38.8 |
| Bax(-) MK-R + NCS | 5.2 | 35.4 | 2.3 | 57.1 |
| P53(-) | 4.7 | 38.3 | 12.7 | 44.3 |
| P53(-) + NCS | 6.4 | 5.6 | 3.6 | 84.4 |
| P53(-) MK-R | 5.7 | 29.9 | 1.7 | 62.7 |
| P53(-) MK-R + NCS | 9.6 | 28.9 | 3.1 | 58.4 |
| P21(-) | 8.0 | 60.5 | 10.7 | 20.8 |
| P21(-) + NCS | 5.6 | 1.6 | 2.0 | 90.8 |
| P21(-) MK-R | 6.9 | 50.9 | 9.6 | 32.6 |
| P21(-) MK-R + NCS | 8.9 | 6.1 | 6.0 | 79.0 |
| Chk2(-) | 5.4 | 52.7 | 4.3 | 37.6 |
| Chk2(-) + NCS | 10.6 | 37.8 | 1.7 | 49.9 |
| Chk2(-) MK-R | 5.1 | 45.1 | 9.2 | 40.6 |
| Chk2(-) MK-R + NCS | 20.3 | 23.3 | 1.8 | 54.6 |

Table S1
